# Convenience and comfort: reasons reported for using drugs alone among clients of harm reduction sites in British Columbia, Canada

**DOI:** 10.1186/s12954-020-00436-6

**Published:** 2020-11-23

**Authors:** Kristi Papamihali, Minha Yoon, Brittany Graham, Mohammad Karamouzian, Amanda K. Slaunwhite, Vivian Tsang, Sara Young, Jane A. Buxton

**Affiliations:** 1 British Columbia Centre for Disease Control, 655 West 12th Avenue, Vancouver, BC V5Z 4R4 Canada; 2 Michael G. DeGroote School of Medicine, McMaster University, 1280 Main Street W, Hamilton, ON L8S 4K1 Canada; 3 School of Population and Public Health, University of British Columbia, 2206 E Mall, Vancouver, BC V6T 1Z3 Canada; 4 British Columbia Centre on Substance Use, 400-1045 Howe St, Vancouver, BC V6Z 2A9 Canada; 5 HIV/STI Surveillance Research Center, and WHO Collaborating Center for HIV Surveillance, Institute for Futures Studies in Health, Kerman University of Medical Sciences, Kerman, Iran; 6 Faculty of Medicine, University of British Columbia, 317 – 2194 Health Sciences Mall, Vancouver, BC V6T 1Z3 Canada

**Keywords:** Using alone, Using drugs alone, Overdose crisis, Overdose, Opioid

## Abstract

**Background:**

North American communities are severely impacted by the overdose crisis, particularly in British Columbia (BC), which has the highest toxic drug overdose death rate in Canada. Most fatal overdoses in BC occurred among individuals using alone and in private residences. This study aimed to assess prevalence and reasons for using drugs alone among people accessing harm reduction services in BC.

**Methods:**

We recruited harm reduction supply distribution site clients from 22 communities across BC. Descriptive statistics and multivariable logistic regression were used to describe factors associated with using alone. Thematic analysis of free-text responses providing reasons for using alone were grouped with survey data and additional themes identified.

**Results:**

Overall, 75.8% (*n* = 314) of the study sample (*N* = 414) reported using drugs alone within the last week. Those that reported using alone did not differ from those that did not by gender, age, urbanicity, or preferred drug use method. Among those that used alone, 73.2% (*n* = 230) used opioids, 76.8% (*n* = 241) used crystal meth, 41.4% (*n* = 130) used crack/cocaine, and 44.6% (*n* = 140) used alcohol in the past week. Polysubstance use involving stimulants, opioids, and/or benzodiazepines was reported by 68.5% (*n* = 215) of those that used alone. Additionally, 22.9% (*n* = 72) of those that used alone had experienced an opioid and/or stimulant overdose in the past 6 months. In a multivariable logistic regression model, having no regular housing and past week crack/cocaine use were associated with using alone (adjusted odds ratio (AOR): 2.27; 95% CI 1.20–4.27 and AOR: 2.10; 95% CI 1.15–3.82, respectively). The most common reason reported for using alone was convenience and comfort of using alone (44.3%). Additional reasons included: stigma/hiding drug use (14.0%); having no one around (11.7%); safety (9.6%); and not wanting to share drugs with others (8.6%).

**Conclusions:**

Using drugs alone, particularly for convenience and comfort, is ubiquitous among people accessing harm reduction services. Overdose prevention measures that go beyond individual behaviour changes, including providing a safer supply of drugs and eliminating stigma, are paramount to mitigate harms. These interventions are especially necessary as emergence of coronavirus disease may further exacerbate unpredictability of illicit drug content and overdose risk.

## Background

The province of British Columbia (BC) has been the epicentre of the overdose epidemic in Canada, experiencing a fivefold increase in illicit drug toxicity deaths between 2010 and 2018. There have been over 5000 overdose deaths in BC since 2016, surpassing annual deaths from motor vehicle incidents and homicides combined [1, 2] and leading to a decline in life expectancy at birth in BC [3]. Furthermore, in 2020, 4 years into the ongoing overdose public health emergency, the declaration of a second public health emergency, due to the novel coronavirus (COVID-19) pandemic, stands to compound adverse effects on people who use drugs (PWUD) who are at risk of overdose [4–6].

The contamination of the illicit drug supply by fentanyl, a synthetic opioid 50–100 times more potent than morphine [7, 8], and more toxic fentanyl analogues has been the primary driver of the overdose epidemic. Since 2017, fentanyl has been detected in more than 80% of drug toxicity deaths in BC [9]. Despite high levels of knowledge of fentanyl risk, PWUD report a lower self-perceived risk of overdose related to fentanyl [10]. PWUD in BC increasingly report knowingly using fentanyl, with two-thirds of those who used drugs alone reporting use of fentanyl [11]. This suggests that people may underestimate their overdose risk which may influence the measures they take to keep safe, including the choice of environment in which they use.

The majority of illicit drug toxicity deaths in BC occur within private residences and among those using alone. In 2019, the place of injury for over half of illicit drug overdose deaths was determined to be in private residences and a further 26.5% in other residences, such as single-room occupancy hotels, shelters, and supportive housing [9]. This is similar to findings in New York City where three quarters of unintentional overdose deaths occurred in a home [12]. Additionally, a closed case report published in September 2018 found that 69% of people who died of an overdose between 2016 and 2017 in BC were using drugs alone [13]. Using alone presents a challenge for overdose response as it eliminates the opportunity to intervene in a timely manner to prevent anoxic brain injury or death.

Public health response to the overdose crisis has largely relied on individual behaviour change to prevent overdose. Public health messaging to prevent overdose encourages PWUD to always use with someone else, to use a small amount of drugs, and to go slowly when using. Additionally, the use of harm reduction services is encouraged, with many services having undergone significant expansion to meet demand. Among others, this includes the BC take-home naloxone (THN) program, expanded opioid agonist treatment (OAT) availability, and observed consumption sites, all of which had a significant impact on preventing overdose deaths [14]. The THN program, for instance, has been responsive to the overdose crisis by getting naloxone kits into the hands of those that need it most [15, 16], with more than 53,000 THN kits reported as used to reverse an overdose between 2017 and 2019 [17]. However, it is important to note that someone else must be present for naloxone to be administered to an individual experiencing an overdose.

Similarly, observed consumption sites, including supervised consumption sites (SCS) and overdose prevention services (OPS), where people use pre-obtained drugs in an observed setting, have been made available in each BC regional health authority [18]. Employment of peer workers at OPS sites has enhanced feelings of comfort and safety among PWUD using OPS sites due to shared lived experience [19, 20]. However, restrictions at observed consumption sites can create barriers for PWUD to access these services. These include limited sites that allow observed smoking, long wait times, crowded environments, and geographic inaccessibility [21, 22]. Furthermore, women may feel safer using alone than in male-dominated OPS site environments [23, 24].

Several studies have explored why people choose the circumstances in which they use drugs, such as in a public place or in a private environment, and with others or alone. Stigma may prevent people seeking health care and lead to non-disclosure of health conditions [25]. Stigma and shame associated with drug use are also commonly cited reasons why people choose to hide their drug use and use alone or in non-public places [26–28]. PWUD also report that using with others often means having to share their drugs, which may be difficult with limited resources to acquire drugs [21, 26, 29]. Additionally, PWUD must currently balance public health recommendations for physical distancing to prevent community transmission of the novel severe acute respiratory syndrome coronavirus 2 (SARS-CoV-2) against recommendations to not use drugs alone.

Understanding factors that influence PWUD to use alone is important in determining effective public health interventions. Previous studies that have investigated why people use alone have often been small qualitative studies, focused on reasons for not using observed consumption sites, and on people who inject drugs in large urban cities [21, 26–29]. To gain a wider understanding of reasons why PWUD use drugs alone, we use data from the Harm Reduction Client Survey administered in 2018 to a diverse sample of PWUD [30]. This study aims to identify prevalence and reasons people report for using drugs alone, and to identify barriers to safer drug use practices in a population who access harm reduction supply distribution sites across BC.

## Methods

### Data collection

Data collection for this study was conducted from May to August 2018 through the 2018 Harm Reduction Client Survey, which was administered at 27 harm reduction supply distribution sites across BC. The methods of site and individual recruitment have been previously described [11, 15, 22].

In summary, a two-stage convenience sampling approach was used to identify participating harm reduction sites who administered the survey over a 2-week period. A total of 37 sites were approached for participation, of which 10 declined to participate due to lack of capacity. Participants were aged 19 years and older, were attending harm reduction supply distribution sites, reported using illicit drugs in the previous 6 months, and were able to provide verbal informed consent at the time of the survey. Participants were compensated $5 CAD for completing the 10-min survey and the harm reduction supply distribution sites were provided an additional $5 CAD per participant for their time and resources.

### Study variables

The primary dependent variable for this study was reporting ‘having used drugs alone’. Participants were asked, ‘In the last 7 days, what percentage of the time did you use drugs alone?’ and answered ‘0%’, ‘1–25%’, ‘26–50%’, ‘51–75%’, ‘76–100%’, or ‘prefer not to say’. For the purpose of this study, the primary dependent variable was grouped into a categorical binary variable: used drugs alone in the last 7 days (indicating any frequency of using drugs alone in the last 7 days) and did not use drugs alone in the last 7 days (indicating never having used drugs alone in the last 7 days). Individuals that did not respond or indicated ‘prefer not to say’ for the dependent variable were treated as missing and excluded from further analysis.

Independent variables of interest included sociodemographic and substance use characteristics. Sociodemographic information included self-identified gender, age, regional health authority of the site, having regular housing (where no regular housing was defined as currently being homeless, having no regular place to stay, having no fixed address, couch surfing, or living in a shelter), having paid employment, having a naloxone kit, and urbanicity. Urbanicity of the site was classified as medium/large urban cities or small urban/rural communities based on a classification system developed by the BC Ministry of Health which combines standard Statistics Canada definitions [31] with index of remoteness, population density, and proximity to urban areas at a Community Health Service Area level [32].

Independent variables related to substance use included preferred drug use method (injection or non-injection), experiencing an opioid or stimulant overdose in the last 6 months, substances used in the last 7 days, and polysubstance use (defined as concurrent use of opioids and stimulants, opioids and benzodiazepines, or stimulants and benzodiazepines). Participants were asked to identify if they had consumed the following substances in the previous week: opioids (including heroin, fentanyl, morphine, oxycodone, methadone, or hydromorphone), crystal meth, cocaine (powder or crack), benzodiazepines, and alcohol. Independent variables where participants did not respond or indicated ‘prefer not to say’ were treated as missing.

Participants were also asked about their reasons for using alone. Respondents that reported using alone at any frequency in the last 7 days were asked, ‘What are some of the reasons you use drugs alone?’ with options including ‘It’s safer to be alone’, ‘It’s more convenient and comfortable to use at home’, ‘I don’t want others to know that I’m using drugs’, ‘Other’, and ‘Prefer not to say’. Participants could select more than one reason if applicable and were prompted to elaborate with a free-text response if they chose ‘Other’.

### Statistical analysis

All analyses were conducted using R version 3.6.1 [33]. Descriptive statistics were used to describe characteristics of the study sample stratified by using drugs alone in the previous week. Chi-square tests of independence were conducted to consider the relation between using alone and other independent variables.

Independent variables with *p* value < 0.25 in bivariate logistic regression analyses with the dependent variable were included in the multivariate logistic regression model [34, 35]. The multivariable model was selected through a backwards selection approach based on the lowest Akaike’s information criteria value [36]. Variables that were not retained in the backwards selection model but that were conceptually relevant, including age and gender, were included in the final multivariable model. Adjusted odds ratios (AOR), 95% confidence intervals (CI), and *p* values were reported for independent variables, where *p* values < 0.05 were considered statistically significant.

Frequency distributions were used to describe the reported reasons for using alone. Where participants indicated ‘other’ as a reason for using drugs alone, free-text responses were assessed using thematic analysis. Themes that were consistent with existing options on the survey were consolidated under that theme; emerging novel themes which did not fit with the existing themes were iteratively reviewed and new themes identified.

### Ethical considerations

Study-related ethics approval was obtained through the University of British Columbia Office of Behavioural Research Ethics (approval number: H07-00570). Site consent and participant verbal informed consent were obtained for survey participation.

## Results

### Characteristics of participants who reported using drugs alone

**Fig. 1:**
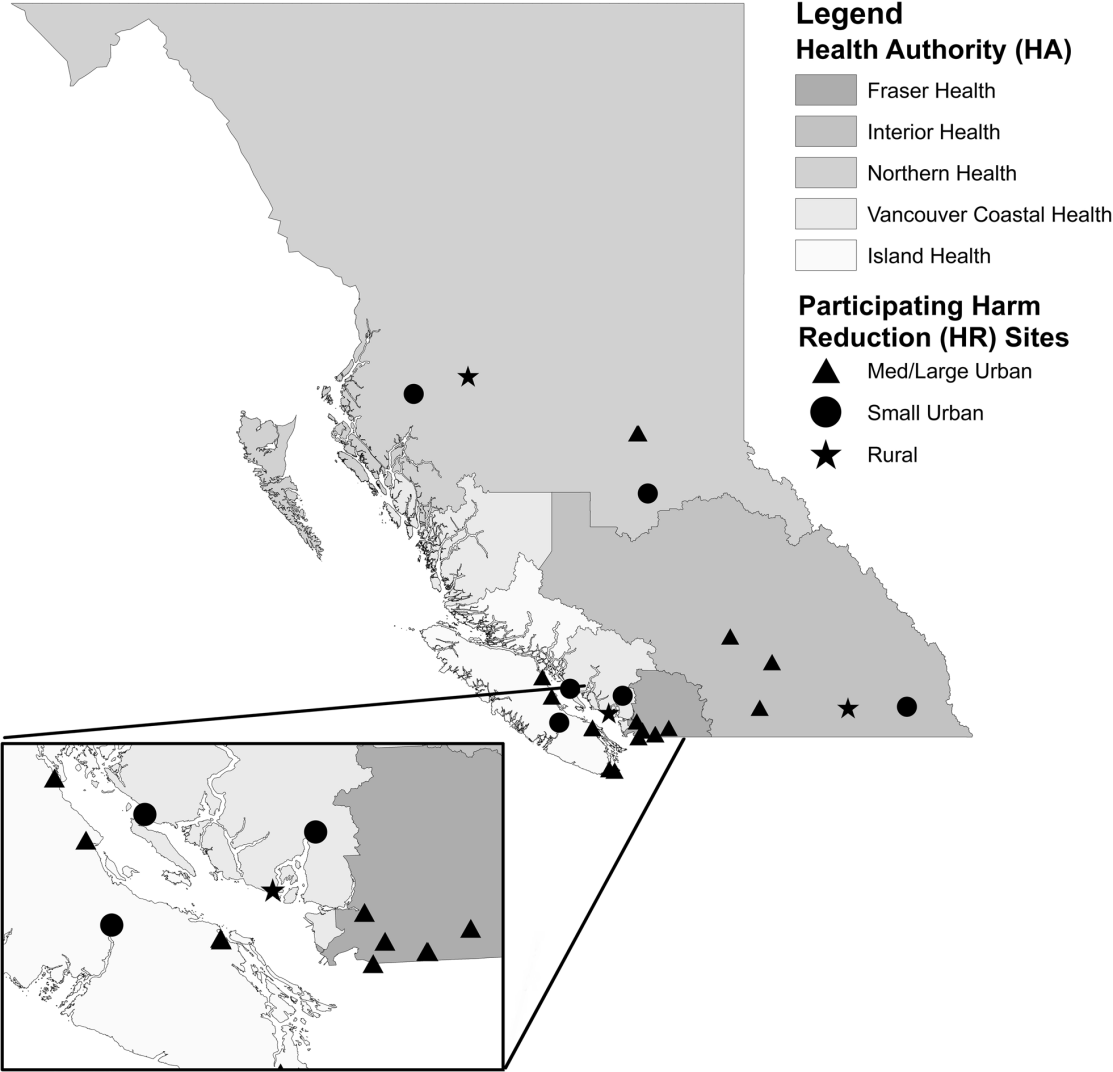
Participating harm reduction supply distribution sites of the 2018 Harm Reduction Client Survey in BC

A total of 486 persons completed the survey across all five geographic health regions in BC. The 27 participating sites were from across BC and varied in urbanicity (Fig. 1). Exclusion of missing responses (*n* = 37) and those who reported prefer not to say (*n* = 35) for the dependent variable provided a study sample of 414.

Table 1 presents the characteristics of the participants. More men (62.6%) than women (34.8%) participated in the survey. Most participants (54.4%) were aged 30–49, while 19.3% were 19–29 and 26.1% were aged 50 years or older. The majority of participants were from medium/large urban areas (72.7%) and from Fraser Health Authority (40.3%). Additionally, most participants were regularly housed (62.8%) but not currently employed (78.5%).

**Table 1:** Summary characteristics of 2018 Harm Reduction Client Survey participants stratified by using alone in the last 7 days

Characteristics	Did not use alone (*N* = 100)	Used alone (*N* = 314)	Total (*N* = 414)	*p* value
	*n* (%)	*n* (%)	*n* (%)	
*Age*				0.16
19–29	16 (16.0%)	64 (20.4%)	80 (19.3%)	
30–39	22 (22.0%)	95 (30.3%)	117 (28.3%)	
40–49	30 (30.0%)	78 (24.8%)	108 (26.1%)	
> 50	32 (32.0%)	76 (24.2%)	108 (26.1%)	
*Gender*^a^				0.16
Woman	40 (40.0%)	104 (33.1%)	144 (34.8%)	
Man	56 (56.0%)	203 (64.6%)	259 (62.6%)	
*Health authority*				0.23
Fraser	38 (38.0%)	129 (41.1%)	167 (40.3%)	
Interior	19 (19.0%)	35 (11.1%)	54 (13.0%)	
Island	23 (23.0%)	68 (21.7%)	91 (22.0%)	
Northern	10 (10.0%)	50 (15.9%)	60 (14.5%)	
Vancouver Coastal (rural communities)	10 (10.0%)	32 (10.2%)	42 (10.1%)	
*Urbanicity*				0.74
Small urban/rural communities	26 (26.0%)	87 (27.7%)	113 (27.3%)	
Medium/large urban cities	74 (74.0%)	227 (72.3%)	301 (72.7%)	
*Currently regularly housed*				< 0.01
Yes	74 (74.0%)	186 (59.2%)	260 (62.8%)	
No	24 (24.0%)	118 (37.6%)	142 (34.3%)	
*Currently employed*				0.19
Yes	23 (23.0%)	53 (16.9%)	76 (18.4%)	
No	75 (75.0%)	250 (79.6%)	325 (78.5%)	
*Naloxone kit possession*				0.61
Yes	66 (66.0%)	213 (67.8%)	279 (67.4%)	
No	30 (30.0%)	85 (27.1%)	115 (27.8%)	
*Preferred drug use method*				0.87
Injection	32 (32.0%)	108 (34.4%)	140 (33.8%)	
Non-injection^b^	59 (59.0%)	191 (60.8%)	250 (60.4%)	
*Opioid use (last 7 days)*^c^				0.04
Yes	60 (60.0%)	230 (73.2%)	290 (70.0%)	
No	36 (36.0%)	83 (26.4%)	119 (28.7%)	
*Crystal meth use (last 7 days)*				< 0.01
Yes	61 (61.0%)	241 (76.8%)	302 (72.9%)	
No	35 (35.0%)	72 (22.9%)	107 (25.8%)	
*Crack/cocaine use (last 7 days)*				0.01
Yes	26 (26.0%)	130 (41.4%)	156 (37.7%)	
No	70 (70.0%)	183 (58.3%)	253 (61.1%)	
*Benzodiazepine use (last 7 days)*				0.29
Yes	10 (10.0%)	46 (14.6%)	56 (13.5%)	
No	86 (86.0%)	267 (85.0%)	353 (85.3%)	
*Alcohol use (last 7 days)*				0.15
Yes	35 (35.0%)	140 (44.6%)	175 (42.3%)	
No	61 (61.0%)	173 (55.1%)	234 (56.5%)	
*Polysubstance use (last 7 days)*^d^				< 0.01
Yes	52 (52.0%)	215 (68.5%)	267 (64.5%)	
No	44 (44.0%)	98 (31.2%)	142 (34.3%)	
*Experienced an opioid or stimulant overdose (last 6 months)*				0.03
Yes	15 (15.0%)	72 (22.9%)	93 (22.5%)	
No	78 (78.0%)	194 (61.8%)	266 (64.3%)	

Missing values account for percentages which do not add up to 100% or total numbers that do not add up to 414

^a^Other genders reported included being a trans man, trans woman, or gender non-conforming which are not shown here due to small sample size (*n* = 9)

^b^Non-injection methods included smoking/inhaling/snorting (56.5%, *n* = 234) and other methods (3.9%, *n* = 16)

^c^Opioids included heroin, fentanyl, morphine, hydromorphone (Dilaudid), methadone, and oxycodone

^d^Polysubstance use was defined as concurrent use of opioids & stimulants, opioids & benzodiazepines, or stimulants & benzodiazepines

Most participants (75.8%) reported using drugs alone in the last 7 days (Table 1), with 47.8% (*n* = 198) reporting using drugs alone more than 25% of the time. Persons who reported using drugs alone did not differ significantly from those that reported not using drugs alone in terms of age, gender, urbanicity, health authority, employment status, having a naloxone kit, or preferred method of drug use. Variables significantly associated (*p* < 0.05) with reporting using alone in the last 7 days compared to those that did not use alone included: not having current regular housing (37.6% vs. 24.0%); experiencing an opioid or stimulant overdose in the last 6 months (22.9% vs. 15%); and reporting use of certain substances, including use of opioids (73.2% vs. 60.0%), crystal meth (76.8% vs. 61.0%), crack/cocaine (41.4% vs. 26.0%), as well as polysubstance use (68.5% vs. 52.0%).

### Factors associated with using drugs alone

Adjusted odds ratios and 95% confidence intervals for factors associated with using drugs alone in the last 7 days are shown in Table 2. Covariates that were not statistically significant in bivariate logistic regression analyses were excluded from the multivariable regression model, except variables that were deemed conceptually relevant (i.e. gender and age), which were forced into the final multivariable model. In the final adjusted model, using drugs alone was significantly and positively associated with not being currently regularly housed (AOR = 2.27 [95% CI 1.20–4.27]) and crack/cocaine use in the last 7 days (AOR = 2.10 [95% CI 1.15–3.82]). Though positively associated with using alone, opioid use, crystal meth use, and experiencing an opioid or stimulant overdose in the last 6 months were not statistically significant.

**Table 2:** Adjusted odds ratios (AOR) and 95% confidence intervals (CI) for odds of using alone versus not using alone

Characteristics^a^	AOR (95% CI)	*p* value
*Age*		
19–29	1.00	
30–39	1.10 (0.47–2.57)	0.82
40–49	0.83 (0.37–1.90)	0.66
> 50	0.91 (0.39–2.12)	0.82
*Gender*		
Woman	1.00	–
Man	1.40 (0.80–2.44)	0.24
*Currently regularly housed*		
Yes	1.00	–
No	2.27 (1.20–4.27)	0.01
*Opioid use (last 7 days)*^b^		
Yes	1.59 (0.87–2.88)	0.13
No	1.00	
*Crystal meth use (last 7 days)*		
Yes	1.58 (0.86–2.88)	0.14
No	1.00	–
*Crack/cocaine use (last 7 days)*		
Yes	2.10 (1.15–3.82)	0.02
No	1.00	–
*Experienced an opioid or stimulant overdose (last 6 months)*		
Yes	1.71 (0.79–3.69)	0.17
No	1.00	–

^a^Final model is based on 306 observations after exclusion of missing responses and responses where ‘prefer not to say’ was indicated for independent variables

^b^Opioids included heroin, fentanyl, morphine, hydromorphone (Dilaudid), methadone, and oxycodone

### Reasons for using drugs alone

Of the 314 participants who reported using drugs alone in the last 7 days, 264 provided reasons for using alone. Of participants that did not provide a reason, 31 indicated ‘prefer not to say’ and 19 did not respond. Additionally, 121 participants selected ‘other’ as a response, of which 113 provided free text with reasons they use drugs alone. Most items that emerged from the free-text responses were synonymous to the initial four options provided and were recoded as such. Two new themes were identified: ‘was alone/had no one to use with’ and ‘do not want to share/cheaper to use alone’. There were an additional 19 miscellaneous responses that could not be coded into one of the six categories.

Reported reasons for using alone are presented in Table 3. Convenience and comfort was the most commonly reported reason for using drugs alone (44.3%; *n* = 139). Additional reasons included: not wanting others to know about drug use or facing stigma around drug use (14.0%; *n* = 44); being alone or having no one else to use with (11.7%; *n* = 37); feeling it was safer to use drugs alone (9.6%; *n* = 30); and not wanting to share with others or feeling it was cheaper to use drugs alone (8.6%; *n* = 27). Reported reasons for using drugs alone did not vary between different genders or age groups, except for not wanting others to know about drug use or facing stigma around drug use which was reported more frequently among those that were less than 50 years old compared to those that were 50 years old or greater (16.9% and 5.3%, respectively, *p* = 0.01).

**Table 3:** Reasons for using drugs alone reported by participants of the 2018 Harm Reduction Client Survey that reported using drugs alone in the last 7 days

Reason for using drugs alone	Participants using drugs alone (*n* = 314) *n* (%)
Convenience and comfort	139 (44.3%)
Don’t want others to know/stigma	44 (14.0%)
*Was alone/had no one to use with*^a^	37 (11.7%)
It’s safer to be alone	30 (9.6%)
*Do not want to share/cheaper to use alone*^a^	27 (8.6%)
*Other reasons*^a^	19 (6.1%)
Prefer not to say	31 (9.9%)
No response	19 (6.1%)

Participants could identify more than one reason for using alone

^a^Reasons identified through thematic analysis of free-text responses

## Discussion

In this study, more than three quarters of participants reported using drugs alone during the previous 7 days. Prevalence of using drugs alone did not differ by age group, gender, geographic region, urbanicity, or preferred drug use method, indicating the pervasiveness of using drugs alone among PWUD accessing harm reduction services. The primary reason participants reported for using drugs alone was convenience and comfort.

To our knowledge, this study is unique in its focus on characterizing and understanding reasons for using drugs alone among a diverse group of PWUD. Previous studies exploring use of drugs alone are limited and mainly qualitative studies that have focused on urban city centres, people who inject drugs, or using drugs alone as it pertains to perceptions around use of OPS and SCS sites [21, 23, 24, 26, 28, 37–40]. In the current study, approximately one-third of the sample was from small urban or rural communities. Additionally, the study included individuals that preferred non-injection methods of substance use, including 56.5% of participants that preferred smoking, inhaling, or snorting.

In this study, we found that comfort and convenience was the most commonly reported reason for using alone, while stigma and not wanting others to know about drug use was reported by 14% of participants. This finding may reflect the study sample, which includes PWUD accessing harm reduction services rather than PWUD that may hide their use and may be more affected by stigma. A previous study conducted by Small et al. [21] showed that one of the factors PWUD take into consideration in their choice of environment is protection from law enforcement and from potential street violence or harassment, which impacts their comfort or anxiety level [21]. Individuals may also have specific rituals and preferences that influence their environment of choice when using substances [22]. Additionally, some participants’ responses implied that they had no alternative but to use alone. Participants reported not having anyone to use with, not having access to an OPS, not being allowed to use in transition home, and experiencing severe pain, further supporting convenience as well as the necessity to use as reasons for using alone.

Our participants also reported that they did not want to or could not afford to share their substances and that it was cheaper to use alone for this reason. In previous studies, PWUD report there is a social expectation to share drugs when using in the presence of others, whether it is from generosity or compensation for providing a safe venue [21, 26, 29].

A portion of participants also identified safety as the reason they used drugs alone, which may result from experiences of violence and harassment and potentially gender-based violence for women who use substances [23, 24, 41]. While prevalence of using alone was high in both men and women and did not differ based on gender in the current study, previous work has highlighted experiences of women using substances alone in order to avoid violent circumstances [23, 24]. This has included reluctance to use OPS and housing-based OPS sites by women who use drugs to ensure personal safety [23, 24]. Response and interventions to prevent use of drugs alone, and thus reduce risk of overdose, must address both the high prevalence of overdose among men that use alone [9, 42], as well as the unique factors that shape women’s experiences, including the disproportionate barriers and burden of overdose among Indigenous women who use drugs [43, 44]. The opening of the first women-only OPS site, SisterSpace, in Vancouver presents one such opportunity to support and provide safe spaces for women who use drugs.

The reasons for using alone described above are particularly salient for people experiencing homelessness and those who have no regular place to stay, which in this study was associated with using alone. People facing homelessness may seek hidden and secluded spaces to use substances to avoid using in public places, where they may face harassment, arrest, or stigma and discrimination [26, 39, 45, 46]. PWUD that are experiencing homelessness must weigh the compounded adverse outcomes that may arise from using substances in public places against the increased risk of overdose faced when using alone [26]. A significant proportion of drug toxicity deaths continue to occur outside, in public buildings, and in temporary residences, such as single-room occupancy hotels, shelters, and supportive housing [9]. Expansion of observed consumption spaces, which see over 60,000 visits a month, has been one approach in mitigating overdose risk among PWUD facing homelessness by providing a safe and private space for substance use where timely response is possible in the event of an overdose.

The use of crack or cocaine in the past week was also associated with using alone. This may reflect perceptions of lower perceived risk of harms when using stimulants, including a lower perceived risk of harm when using both opioids and stimulants [24, 47]. Opioid use, crystal meth use, and polysubstance use were highly prevalent among participants that used substances alone. Furthermore, 23% of those that used alone had experienced an opioid or stimulant overdose in the past 6 months. Considering the highly toxic drug supply in circulation in North America, and the compounded adverse outcomes of concurrently using opioids and stimulants, or opioids and benzodiazepines [48], using alone presents an added risk with no potential for response in the event of an overdose. In addition, smoking of substances is not permitted at most SCS and OPS sites, which may also push people who smoke drugs to not attend observed consumption sites and thus use alone.

While 67.4% of participants that reported using alone had a naloxone kit, the opportunity to respond may not be possible if using alone. However, some PWUD may inform friends or neighbours they are going to use, leave their door unlocked, and ask that they check on them [22]. Similarly, McLean details occurrences of people who ‘overdosed while using alone, yet were saved by friends or family who were somehow alerted to the danger’, such as hearing a thud [49]. Thus, having a naloxone kit, even if using alone, is important to allow quick intervention and could allow a bystander to respond to an overdose.

Public health messaging that urges individuals to avoid using drugs alone and to use in observed consumption spaces is important and provides options for PWUD to be safer in their drug use. OPS and SCS sites are an effective intervention for some PWUD, and eliminating stigma associated with drug use remains imperative in allowing people to feel safe and comfortable accessing these services. However, there are complex environmental, social, and personal factors that influence the choice (or lack thereof) to use alone. Individuals use alone despite knowing fentanyl is in the drug supply [11], and despite having had previous overdoses. Therefore, along with providing a wide spectrum of overdose prevention interventions, such as distribution of naloxone, drug testing services, and observed consumption sites, it is also important to implement interventions that do not rely solely on individual behaviour changes but rather address the source of the opioid overdose epidemic—the toxic illicit drug supply.

Providing a safer supply of drugs can offer added safety by allowing PWUD to be aware of the substance they are using and the amount they are taking [50–53]. To enable people to isolate or quarantine in place in the context of the dual public health emergencies of the COVID-19 pandemic and the overdose crisis, Health Canada issued temporary revisions to the Controlled Drugs and Substances Act to allow pharmacists to extend, transfer, and deliver prescriptions of controlled substances. Provision of prescribed opioid alternatives was approved in BC; however, barriers, including a limited number of healthcare providers willing to prescribe, pose a challenge in uptake [54]. PWUD have been doubly impacted by the dual public health emergency considering increased susceptibility to transmission of SARS-CoV-2 due to the presence of underlying health concerns [6], close living conditions among those that are precariously housed, and difficulties in accessing needed supplies, substances, and medications while physical distancing. Additionally, recommendations for physical distancing have sometimes been interpreted at odds with recommendations to not use drugs alone [55]. While the full effect that the COVID-19 pandemic has had on PWUD is yet to be seen, there has been an increase in overdose deaths in BC since in March 2020 [1]. Moreover, while observed consumptions sites were deemed essential services and remained open throughout the duration of the pandemic, there has been a decrease in visits at observed consumption sites [2]. Balancing recommendations to not use alone and recommendations to self-isolate presents an additional challenge for PWUD and further highlights complex factors that influence individual drug use patterns.

Our study examined the reported reasons people use alone using data collected from a survey, but it does not explore the context and perspectives of the respondents in depth. However, as most previous studies have been qualitative, our study adds to existing knowledge by comparing and quantifying key reasons identified for using alone among PWUD accessing harm reduction services. As this study relied on self-reported substance use patterns, some responses may be subject to social desirability bias and may not accurately represent behaviours. It should also be noted that our sample consisted of PWUD who attended harm reduction sites, suggesting that they are to some degree already connected with harm reduction services and programs. Thus, the findings of this survey may not be generalizable to all those who use substances in BC. People who are more affected by stigma and hide their use may not use harm reduction services and thus not participate in the survey. Nonetheless, this study captured reasons reported for using alone from a large and diverse sample of PWUD who differed in terms of drugs used, route of drug administration, geography, and demographics.

## Conclusion

We found the majority of PWUD that attended harm reduction sites across the province reported using drugs alone in past week for a variety of reasons, but in particular convenience and comfort. Efforts to prevent overdose deaths among PWUD must take into consideration the reasons why people use drugs alone in order to support harm reduction measures that are pragmatic and effective. In addition to providing a spectrum of harm reduction services and addressing stigma associated with drug use, interventions that address the toxic illicit drug supply, whose unpredictability is likely exacerbated by the impact of COVID-19, are needed to support people who use drugs to be safe, even when using alone. Therefore, access to a safer supply of drugs is paramount.

## Data Availability

The datasets generated and/or analysed during the current study are not publicly available due them containing information that could compromise research participant privacy/consent, but are available from the corresponding author on reasonable request.

## Abbreviations

AOR: Adjusted odds ratio
BC: British Columbia
COVID-19: Coronavirus disease 2019
OAT: Opioid agonist treatment
OPS: Overdose prevention services
PWUD: People who use drugs
SARS-CoV-2: Severe acute respiratory syndrome coronavirus 2
SCS: Supervised consumption site
SUAP: Substance use and addictions program
THN: Take-home naloxone
